# Flavoromics Approach in Critical Aroma Compounds Exploration of Peach: Correlation to Origin Based on OAV Combined with Chemometrics

**DOI:** 10.3390/foods12040837

**Published:** 2023-02-16

**Authors:** Qianqian Li, Bei Li, Rong Zhang, Shuyan Liu, Shupeng Yang, Yi Li, Jianxun Li

**Affiliations:** 1 Key Laboratory of Agro-Products Quality and Safety Control in Storage and Transport Process, Ministry of Agriculture and Rural Affairs, Institute of Food Science and Technology, Chinese Academy of Agricultural Sciences, Beijing 100093, China; 2 Key Laboraory of Tropical Fruits and Vegetables Quality and Safety for State Market Regulation, Hainan Institute for Food Control, Haikou 570314, China

**Keywords:** peach, HS-SPME/GC-MS, critical aroma compounds, OPLS-DA, SUS plots

## Abstract

It is essential to seek the critical aroma compounds to identify the origins of peach as well as provide a guidance for quality evaluation. In this study, the peach was characterized by HS-SPME/GC-MS. Subsequently, the odor activity value (OAV) was calculated to specify the primary aroma-active compounds. Afterwards, the chemometrics methods were employed to explore the potentially critical aroma on the basis of *p* value, fold change (FC), S-plot, jack-knifing confidence interval, variable importance for projection (VIP), and the Shared and Unique Structures (SUS) plots. As a result, five compounds (methyl acetate, (E)-hex-2-enal, benzaldehyde, [(Z)-hex-3-enyl] acetate, and 5-ethyloxolan-2-one) were considered as critical aromas. Moreover, the multi-classification model was developed with an outstanding performance (accuracy of 100%) using the five critical aroma. Moreover, the potential chemical basis of odors was sought through sensory evaluation. In addition, this study provides the theoretical and practical foundation for geographical origin traceability and quality evaluation.

## 1. Introduction

The peach (*Prunus persica* L. Batsch) has been cultivated for more than 3000 years in China [1]. It is known that the germplasm resources of peaches are abundant throughout the world, which is attributed to its long history of cultivation and wide geographic distribution. The peach is ranked as one of the most nutritionally and economically valuable fruits due to its widespread consumption worldwide [2]. Moreover, the peach qualifies as a healthy food in view of high nutrients, low calories, as well as an ample amount of vitamins and minerals [3]. To a certain extent, the peach provides health benefits by improving digestion, reducing cholesterol, and boosting the immune system, which is particularly dependent upon the high content of dietary fiber, antioxidants (vitamins C, vitamins A, polyphenols), calcium, and potassium [4]. Aroma is one of the essential factors for evaluating the fruit quality [5]. It is reported that the unique aroma compounds in peach are C6 compounds, lactones, aldehydes, alcohols, esters, and so on [6,7]. Typically, the aroma is contributed by a multitude of aroma-active chemicals with the concentrations over the odor threshold [8,9]. Definitely, the OAV is essential to assess the contributions of volatile compounds to the aroma [10]. As the aroma could provide fingerprint information on its geographical origin, it is imperative to search the critical aroma compounds connected with each geographic region, which could not only identify the origin of peach but also provide guidance to reduce the denaturation or damage to the flavor-characterizing compounds and active ingredients of the fruit.

Generally speaking, gas chromatography-mass spectrometry (GC-MS) is the frequently used method for volatile compound separation and quantification in fruit [11]. As a rapid volatile extraction technique, headspace-solid phase microextraction (HS-SPME) has the advantages of simple, quick, solvent-free treatment and green environmental protection [12,13,14]. The chemometrics methods are powerful approaches to mine data thoroughly, develop efficient and robust models, and give a rational interpretation of complex data sets [15]. Principal components analysis (PCA) [16,17], partial least squares-discrimination analysis (PLS-DA) [18,19] and orthogonal partial least squares-discrimination analysis (OPLS-DA) [20] are powerful tools to explore the critical aroma compounds. Moreover, the Shared and Unique Structures (SUS) plots generated using OPLS-DA are firmly established as a forceful tool to investigate the shared and unique structures of multiple classification models [21].

In this study, the volatile profiles were characterized using HS-SPME/GC-MS. Subsequently, the volatile contribution of OAV was calculated to specify the primary aroma-active compounds. After that, the chemometrics methods were carried out to reveal the differences and explore the statistically and potentially significant aroma compounds among the three origins on the basis of S-plot analysis, significant difference, fold change (FC), and jack-knifing confidence intervals. In addition, the SUS plots were performed to give a visualization of the shared and unique biomarkers among multiple groups. Finally, the critical compounds through the two strategies of OAV and chemometrics were employed to develop the multi-classification models and to explore the potential chemical basis of odors using sensory evaluation.

## 2. Materials and Methods

### 2.1. The Peach Samples Collection

The peach (*Prunus persica* (L.) Batsch) samples were acquired from three characteristic provinces of Beijing (BJ), Shandong (SD), and Hebei (HB) in northern China in 2021. A total of 144 samples were collected from 18 plantation bases of BJ, SD, and HB, that is, 48 samples were collected from six bases for each province. In order to make the results more representative, four samples were mixed as one for determination. The mature peach samples were gathered according to the standard of GB/T 8855-2008 in China. The samples were stored at 4 °C and analyzed within 24 h after collection.

### 2.2. The Peach Samples Preparation for Volatile Compounds Analysis

#### 2.2.1. Extraction of Volatile Compounds

The extraction of volatile compounds from the peach samples was performed using the HS-SPME technique which was equipped with carboxen/polydimethylsiloxane fused silica (CAR/PDMS) coated fiber (30 × 0.25 × 0.25) of 2 cm (Supelco, Inc., Bellefonte, PA, USA). A mixture of 3 g peach sample, 0.8 g NaCl, 2 mL distilled water, and 20 uL internal standard of 2-butanol, were placed into a 20 mL vial with a magnetic rotor, and sealed with silicone septa (Sigma Chemical Co., St. Louis, MO, USA) to incubate at 50 °C. After 20 min of equilibration, the CAR/PDMS coated fiber was exposed in the headspace, placed for 40 min before being inserted into the GC-MS injector port at 250 °C for 3 min.

#### 2.2.2. Volatile Compounds Detection

The volatile compounds were identified using GC-MS (Agilent 7890A GC and Agilent 7000B mass selective detector) with the HP-5MS capillary column (30 m × 0.25 mm × 0.25 µm) for separation. The highly pure helium (≥99.99%) was utilized as the carrier gas and the flow rate was set as 1 mL/min. The temperature program of the GC oven was optimized as follows: 40 °C, kept for 3 min, rise to 180 °C at 5 °C/min, held for 3 min, increased to 300 °C at 20 °C/min, held for 3 min. The ionization method was electron ionization (EI). The MS was scanned in an EI mode of 70 eV over the mass range of 40 to 500 with source temperature set as 230 °C and transfer line temperature set as 250 °C. The 2-butanol (10 μL, with a final concentration of 2.70 mg L^−1^) was set as an internal standard for semi-quantitative analysis [22].

#### 2.2.3. Volatile Compounds Identification

Prior to mass spectral matching, the peaks were analyzed using the deconvolution approach (TraceFinder 5.1) on AMDIS software (www.amdis.net accessed on 1 August 2022). Moreover, the volatile compounds were identified through the NIST library combined with the retention index (RI), as reported. As for the compound identification, the compound was selected according to the minimum ΔRI (the calculated RI minus the library RI). Strictly speaking, a sequence of *n*-alkanes (from C_7_ to C_40_) was adopted as external references to calculate the RI for each volatile. The RI was calculated according to Equation (1), where *n* was the number of carbon atoms of normal alkanes, *t*_a_ was the retention time of the volatile compound, *t_n_* and *t_n_*_+1_ were the retention time of *C_n_* and *C_n_*_+1_, respectively.

(1) $$RI=100\times n+100\times \frac{t_{a}-t_{n}}{t_{n+1}-t_{n}}$$

The identified volatile compounds were semi-quantified using 2-butanol as an internal standard with Equation (2), where *A_c_* and *A_i_* were the peak areas of the volatile compound and internal standard, respectively. The concentration of internal standard was *C_i_*.

(2) $$C=\frac{A_{C}}{A_{i}}\times C_{i}$$

### 2.3. OAV Calculation of the Aroma Compounds

The contribution of aroma compounds was estimated using the OAV. The OAV of the aroma compounds was calculated as the ratio of concentration to the odor threshold [23]. The aroma compounds with OAV > 1 were considered as contributing greatly to the aroma profile. It is worth noting that the OAVs presented in this study have some limitations since the odor threshold values were calculated in water. Although it was not the real situation for peach, it could reflect the OAVs of volatile compounds to some extent, as more than 80% of the peach pulp was water.

### 2.4. Sensory Evaluation

Aroma profiling was carried out using ten assessors (five females and five males, aged from 22 to 30) that had rich experience in sensory evaluations and quantitative description analysis. The assessors evaluated the aroma, gave comments, and scored the peach samples according to the standard of GB12313-1990 in China for traditional sensory evaluation. The traditional sensory evaluation was conducted three times in parallel. Subsequently, the aroma terms were sought to describe the aroma characteristics according to the standard of GB/T10221-2021 in China from the traditional sensory evaluation. On the basis of relevant literature [24], the inexact words were deleted. Ultimately, four sensory attributes and their definitions of peach samples were established, which were demonstrated as follows: methyl acetate for ‘sweet, fruity-like’ note, [(Z)-hex-3-enyl] acetate for ‘green and grassy’ note, hexyl acetate for ‘fruity’ note, and nonan-1-ol for ‘floral’ note.

Based on the established aroma terms, ten assessors evaluated the peach on a digital scale of 0 to 5. In particular, the intensity scales ranged from 0 to 5 for non-existent, just detectable, weak, medium, strong, and very strong, respectively. The samples were in randomized order for three replications.

### 2.5. Statistic Analysis

The OPLS-DA analysis of HS-SPME/GC-MS data was performed on SIMCA 14.1 (Umetrics, Umeå, Sweden); the PCA analysis was carried out on MatLab (Version 2021b, Natick, MA, USA). The HS-SPME/GC-MS data were preprocessed using pareto scaling (Par) before multivariate analysis. The Par is calculated as Equation (3), where $\bar{x}$ denotes the mean value of variables. For the OPLS-DA model, R^2^ and Q^2^ indicate the model developed and predicted ability, respectively. The closer the two parameters get to 1, the better is the performance of the model.

(3) $$P\mathrm{ar}=\frac{x-\bar{x}}{\sqrt{SD}}$$

As an unsupervised multivariate method, PCA is a frequently-used chemometrics tool to reduce the dimension of complicated data to the subspace with fewer principal components (PCs). PCA is adept at revealing the underlying pattern of a dataset in order to seek the similarities and differences. In this work, PCA was utilized to reduce the dimensions and to observe a primary assessment of the variation between classes through the HS-SPME/GC-MS datasets, with Par pretreatment.

The PLS-DA approach is a supervised multivariate statistical method [15], which decomposes the *X* matrix into the categorical response of *Y* as described in Equation (4),

(4) $$X=TP_{}^{T}+E$$

The OPLS-DA approach is a modification of the PLS-DA method [20], which decomposes the *X* matrix into three parts according to the categorical response of *Y* as described in Equation (5),

(5) $$X=T_{P}P_{P}^{T}+T_{O}P_{O}^{T}+E$$

where *T_P_* and *P_P_* are the score and loading of *X* matrix for prediction, respectively. Likewise, *T_o_* and *P_o_* are the score and loading that is orthogonal to Y. E is the residual matrix of X. Namely, the systematic variation of X is divided into three parts of Y-predictive, Y-orthogonal, and the residual matrix.

The volatile compounds were analyzed on SPSS 26.0 (SPSS Inc., Chicago, IL, USA) for *t*-test with the significant difference being *p* < 0.05. When measuring the content of volatile compounds, three replicates were employed, and the results were based on three replicates expressed as the mean ± standard deviation. 

## 3. Results and Discussion

### 3.1. Characterization the Profile of Volatile Components by HS-SPME/GC-MS

The peach samples from three cultivation regions of BJ, SD, HB were analyzed using HS-SPME/GC-MS. The volatile compounds of peach from the cultivation regions are shown in Table 1. As demonstrated in Table 1, a total of 23 volatile compounds were identified using AMDIS through the deconvolution approach and ΔRI included seven esters, five alcohols, three hydrocarbons, two aldehydes, two benzenes, two ketones, one lactone, and one ether. In fact, the identified volatile compounds number was 17, 17, and 15 for BJ, SD, HB, respectively, which accounted for 70.83%, 73.91%, and 71.43% of the total volatile compounds with the exclusive volatile compounds observed in one or two cultivation regions. Strictly speaking, the volatile compounds of pentan-3-one, methyl hexanoate and phenylmethanol were the specific compounds of BJ, HB, and SD, respectively. In addition, esters, alcohols, and aldehydes were the main volatiles in peach according to the number of compounds. As shown in Table 1, the volatile compounds distribution for the peaches from different cultivation regions were different. Some compounds were in extremely high concentrations among the three planting regions, i.e., benzaldehyde, hexyl acetate, and (Z)-hex-2-enyl acetate (>1500 µg/kg). As for the volatile compounds, some exhibited notably high concentrations (>1500 µg/kg) in individual areas, such as [(Z)-hex-3-enyl] acetate was high only in BJ and (E)-hex-2-enal was high only in SD. Yet, some compounds were distinct among the three groups; for instance, the methyl acetate was significantly higher in SD, which was almost six times as much as BJ and HB, and the concentration of ethylbenzene in BJ was twice as much as SD and HB. In brief, the volatile compounds from BJ, SD, HB have been characterized, and they will be the inputs for the following OAV and chemometrics analysis to explore the critical compounds.

### 3.2. Critical Aroma Compounds Exploration from the Perspective of OAV

The contribution of individual volatile compounds was evaluated using OAV upon taking its concentration and the corresponding odor threshold into account. Namely, a higher OAV was positively associated with a stronger aroma. The volatile compounds with OAV > 1 were specified as primary aroma compounds. The OAV of volatile compounds which were accompany with an odor description and threshold values are shown in Table 2. A total of seven aroma compounds including three esters of hexyl acetate, [(Z)-hex-3-enyl] acetate and methyl acetate, two aldehydes of (E)-hex-2-enal and benzaldehyde, one alcohol of nonan-1-ol, one lactone of 5-ethyloxolan-2-one were considered as primary aroma-active compounds with OAV > 1 (Table 3), which is consistent with a previous study [23].

Generally, the esters of hexyl acetate and [(Z)-hex-3-enyl] acetate with relatively high OAV values, contributed to ‘fruity’ and ‘green and grassy’ odor notes [25]. In addition, the methyl acetate had ‘sweet and fruity’ odor properties. The three esters were regarded as primary contributors to the peach [26]. The substrates for esters were afforded via the lipoxygenase (LOX) biosynthetic pathway [27]. The alcohol acyl transferase (AAT) was a critical enzyme that catalyzed the conversion of alcohols into aliphatic esters. As for the (E)-hex-2-enal, it presented the notes of ‘green and grassy’ [28]. Admittedly, the unsaturated fatty acids were oxidized into various C6 aldehydes during the homogenization process. Specifically, the (E)-hex-2-enal and nonan-1-ol derived from linoleic acid and linolenic acid underwent dioxygenation of a lipoxygenase-catalyzed reaction by LOX [29]. The nonan-1-ol with the odor characteristics of ‘floral and rose’ was yielded using hydroperoxide lyases (HPL) acting on hydroperoxides, which was metabolized to alcohol using alcohol dehydrogenase (ADH) [30]. As for 5-ethyloxolan-2-one, it contributed to the ‘sweet and coconut’ odors for peach samples. The 5-ethyloxolan-2-one was derived from the fatty acid metabolism through the β-oxidation pathway [31], which was yielded by means of a series of procedures of dehydrogenating, epoxidizing, hydroxylating, shortening by β-oxidation, and esterifying by hydroxyacetyl-coenzyme A [32]. In addition, benzaldehyde, responsible for ‘sweet’ notes [33], gives the ‘sweet’ flavor for the peach. As a matter of fact, the benzaldehyde, originated from amino acids of phenylpropanoid compounds [34], was synthesized from phenylalanine using aromatic amino acid decarboxylases. Moreover, the phenylalanine was biosynthesised through shikimate or arogenate pathways, and deaminated via the enzyme of phenylalanine ammonialyase [31]. In the previous study, we speculated that the generation of benzaldehyde might be associated with the enzyme of chorismate synthase [35].

As could be seen from Table 3, the richness of the aroma characteristics were different among the three cultivation regions, which was mainly due to the different combinations of the primary aroma compounds and the distinct contributions of each compound endowed with unique odors. Consequently, seven aroma compounds of methyl acetate, (E)-hex-2-enal, benzaldehyde, [(Z)-hex-3-enyl] acetate, 5-ethyloxolan-2-one, nonan-1-ol, and hexyl acetate were screened and ascertained as the primary aroma-active compounds of the three cultivation regions of BJ, SD, HB, in accordance with the OAV evaluation.

### 3.3. Critical Aroma Compounds Exploration from the Perspective of Chemometrics Methods

#### 3.3.1. Volatile Components Modeling with PCA and OPLS-DA Approaches

The PCA algorithm, one of the most frequently used chemometrics approaches, was used to project the HS-SPME/GC-MS data set to a lower dimensional space and to compare the differences among the three groups. To develop the PCA model, the first two PCs were selected with a total variance of 84.4%. Specifically, the first principal component (PC1) accounted for 50.9% of the total variance while the second principal component (PC2) contributed 33.5%. The overview of the two-dimensional score (Figure S1) plot was clearly segmented, which implied that there were indications of grouping among different cultivation regions. Moreover, the score plot also reflected the impact of cultivation regions on peach aroma composition. The results indicated that BJ and HB had tiny difference according to PC1, while they could be separated along the PC2 dimension. In contrast, there were striking differences between SD vs. BJ as well as SD vs. HB along the PC1 dimension. It could be concluded that the discrepancy between SD vs. BJ and SD vs. HB were significantly greater than that between BJ vs. HB. It might be deduced that HB vs. BJ were relatively near to each other compared with SD. This result suggests that the identified aroma using the HS-SPME/GC-MS-based method could be utilized in peach discrimination. To uncover the underlying difference among BJ, SD, and HB, the supervised method OPLS-DA was applied.

**Figure 1:**
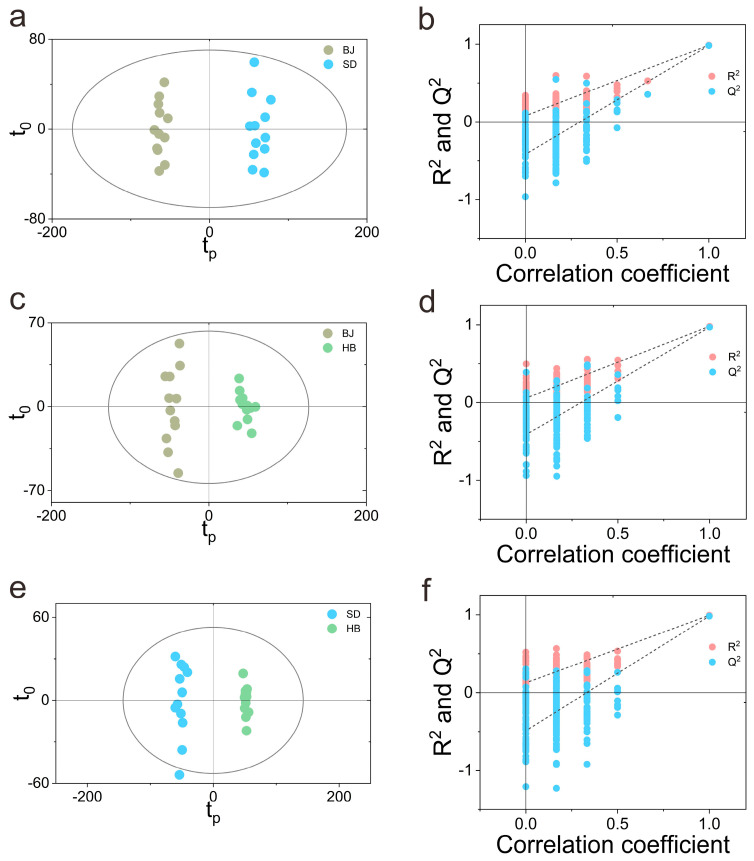
The OPLS-DA analysis of the HS-SPME/GC-MS data. (**a**) score plot for BJ vs. SD; (**b**) validation plot of 200 permutation tests for BJ vs. SD; (**c**) score plot for BJ vs. HB; (**d**) validation plot of 200 permutation tests for BJ vs. HB; (**e**) score plot for SD vs. HB; (**f**) validation plot of 200 permutation tests for SD vs. HB. The slope of R^2^ > 0 together with the intercept of Q^2^ on the *y*-axis < 0 indicating a valid model.

In order to discover the differential critical aroma among the three cultivation regions of the three pair-wise models of BJ vs. SD, BJ vs. HB, SD vs. HB, the supervised chemometrics methods of OPLS-DA was performed for pairwise comparison. In addition, the permutation test was carried out to estimate whether the performance of the OPLS-DA model was acceptable [36] by comparing the multiple random permutated *y* variables and the original *y* variables through random sampling. Seven-fold cross-validation was executed to verify the accuracy and reliability of the model [37] on the three pair-wise models with the evaluation parameters of the total explaining the X-variance R^2^(X), the model developing the ability of R^2^(Y), and the cross-validated predictive ability Q^2^(Y). As for the three models (Figure 1a–f), they were all established with one predictive and one orthogonal component. The R^2^(Y) and Q^2^(Y) for the three models were all satisfied with R^2^(Y) > 0.980 and Q^2^(Y) > 0.969. Actually, the permutation test was connected with the slope of regression plot and the intercept on the *y* axis. A greater slope together with a smaller intercept would yield a more reliable model with better quality and predictive ability. The results of the permutation test with 200 iterations for the three models are demonstrated in Figure 1 and Table 4. Moreover, the Q^2^ and R^2^ for the three OPLS-DA models were all higher than the permutation tests (Table 4), which indicate that the models were suitable for discriminating and seeking potentially critical aromas. The results of the three permutation tests revealed that the classification models were not over-fitting [38]. As postulated above, the three robust OPLS-DA classification models were developed with an acceptable separation ability for exploring the potential critical aroma compounds.

#### 3.3.2. Critical Aroma Components Exploration by S-Plot, Jack-Knifing Confidence Interval, VIP, *t*-Test, and FC

**Figure 2:**
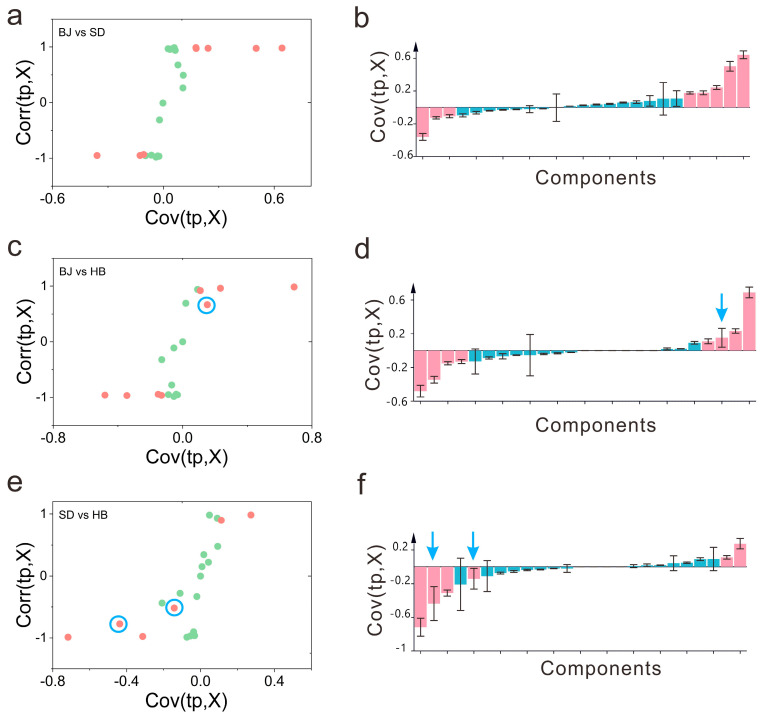
The S-plot and loading plot with jack-knifed confidence intervals. (**a**) S-plot of BJ vs. SD; (**b**) loading plot of BJ vs. SD; (**c**) S-plot of BJ vs. HB; (**d**) loading plot BJ vs. HB; (**e**) S-plot of SD vs. HB; (**f**) loading plot of SD vs. HB. The highlighted pink solid circle in S-plot represents the potential metabolites (|p(cov)| ≥ 0.1 and |p(corr)| ≥ 0.5); the highlighted pink rectangle with confidence interval represents the corresponding potential aroma in S-plot; the blue arrow in the loading plot represents the compound with high confident interval; the blue circle in the S-plot represents the corresponding compound in the loading plot.

To reveal the potential differential aroma compounds among the three cultivation regions, the following restrictions were taken into consideration. Firstly, the S-plot together with the jack-knifed confidence intervals were employed for potential critical aroma exploring [21]. Secondly, the VIP, expressing the contribution of each variable, was utilized to seek the potential aroma with the threshold greater than 1 [39]. Thirdly, the potential compounds were characterized on the basis of the *p* values (<0.05) and FC (|log_2_ (FC)| > 1). The S-plot manifested the covariance and the correlation between the X variables and the predictive score [21]. Both the contribution (covariance, Cov(*t*, X)) and the reliability (correlation, Corr(*t*, X)) are shown in the scatter plot. As a matter of fact, the two aspects of high magnitude and high reliability acted together to demonstrate a low risk of spurious correlations. The calculation of Corr(*t*, X) corresponded to Cov(*t*, X) and the standard deviation of the *t* and *X* variable ($C\mathrm{orr}(t,x)=\frac{Cov(t,X)}{\sigma_{t}\sigma_{x}}$). In this study, the criteria for the S-plot was set with |p(cov)| ≥ 0.1 and |p(corr)| ≥ 0.5. The visualization of candidate aroma compounds to distinguish the pair-wised comparison of BJ vs. SD, BJ vs. HB, SD vs. HB together with the jack-knifing confidence intervals are demonstrated in Figure 2. The jack-knifing confidence interval ($CI=SE_{CV}t(\alpha ,df)$) was determined using SE_CV_, the standard error for each loading, *t*, the statistical value with α of 0.05, and *df*, the degrees of freedom.

As shown in Figure 2a of the S-plot, eight compounds (solid pink circle) were identified as the potential differential compounds that were responsible for the paired comparison of BJ and SD. In the loading plot (Figure 2b), the highlighted potential critical aroma compounds were matched with the S-plot one-by-one. As demonstrated in Figure 2a,b, the confidence interval for all potentially critical aroma compounds in the loading plot was relatively low, which indicated that all the eight potentially critical aroma compounds were located in credible regions. With regards to the VIP, four compounds were filtered as candidates to characterize the differences with VIP > 1 (Table S1). As a consequence, four compounds of methyl acetate, benzaldehyde, (E)-hex-2-enal, and [(Z)-hex-3-enyl] acetate were considered as the potential critical compounds for BJ and SD by taking a comprehensive consideration of the evaluation criterion, including *p*-values and FC. Analogously, as for the paired comparison of BJ and HB, eight compounds were screened as potentially critical compounds by means of the S-plot (Figure 2c). It can be deduced that the 1,2-xylene (with blue arrow) might give an ambiguous response for the two discriminated groups (Figure 2d). In addition, the raw data (Figure S2a) further confirmed that the two regions were slightly overlapped. As far as the VIP, four compounds were found as critical aroma compounds (Table S1). On the whole, four compounds of benzaldehyde, (E)-hex-2-enal, [(Z)-hex-3-enyl] acetate, and 5-ethyloxolan-2-one were regarded as the potentially critical aroma compounds to distinguish BJ and HB using S-plot, jack-knifing confidence, VIP, *p* values and FC. Likewise, for the pair-wise comparison of SD and HB, six compounds were screened using the S-plot (Figure 2e). It could be speculated that the benzaldehyde and phenylmethanol (blue arrow) with large confidence intervals might be situated in uncertain regions (Figure 2f). However, the raw data (Figure S2b,c) ascertained the benzaldehyde did not seem to be vital. Table S1 details the identified discriminant aroma with the relative influence of VIP values on the model. Accordingly, four aroma compounds were considered as candidate critical aroma compounds. After taking *p* values and FC into account, three aroma compounds of methyl acetate, (E)-hex-2-enal, and 5-ethyloxolan-2-one were ultimately determined as the potential critical compounds to distinguish SD and HB.

To sum up, the critical aroma components of the three groups of BJ vs. SD, BJ vs. HB, and SD vs. HB were ascertained with an S-plot together with the jack-knifed confidence intervals and VIP through the paired OPLS-DA approach in cooperation with the *p* values and FC. Overall, the five primary aroma compounds methyl acetate, (E)-hex-2-enal, benzaldehyde, [(Z)-hex-3-enyl] acetate, and 5-ethyloxolan-2-one were specified as differential biomarkers for the three groups. 

#### 3.3.3. Critical Compounds Characterization upon the Strategy of SUS-Plot

SUS-plot, the scatter plots for correlation from the predictive component of Corr(*t*p, X) derived from two individual OPLS models, was produced to give a visualization to determine the shared and unique structures of multiple groups [40]. It was of great interest to find both shared and unique compounds among the multi-model for an overall understanding of the difference as a comparison of the models [21]. Specifically, the compounds on the diagonal represented the shared compounds that had the same effect in both models. Alternatively, the compounds that were located far away from the diagonal symbolized the unique compounds that were specific to either of the two models.

**Figure 3:**
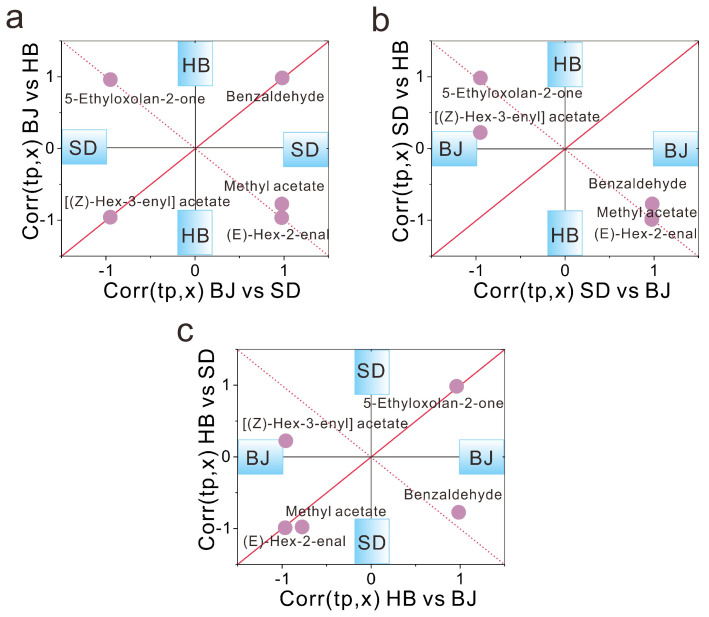
The SUS-plots among BJ, HB, and SD. (**a**) BJ vs. SD and BJ vs. HB; (**b**) SD vs. BJ and SD vs. HB; (**c**) HB vs. BJ and HB vs. SD. The aroma compounds close to the diagonal line are equally affected in both lines and considered as common potential biomarkers; the aroma compounds close to the *x* axis are specific for cultivation in *x* axis and close to *y* axis are specific for cultivation in *y* axis.

It is worth mentioning that the five compounds acted as the critical potential aroma for three paired comparisons of BJ vs. SD and BJ vs. HB, SD vs. BJ and SD vs. HB, HB vs. BJ and HB vs. SD. To obtain a clear overview on the SUS plots, the five aroma compounds were visualized, see Figure 3. The SUS-plot of the two models of BJ vs. SD and BJ vs. HB are demonstrated on Figure 3a. As seen from Figure 3a, all the five potential crucial compounds were regarded as the shared critical aroma along the diagonal line of SD and HB. With respect to the two models of SD vs. BJ and SD vs. HB (Figure 3b), the shared aroma compounds of HB and BJ spanned four compounds (methyl acetate, (E)-hex-2-enal, benzaldehyde, 5-ethyloxolan-2-one), while one ([(Z)-hex-3-enyl] acetate) was ascribed as a unique feature (concentration significantly higher) to BJ with the *p*(corr) close to 0 for HB. As far as the models of HB vs. BJ and HB vs. SD (Figure 3c), four critical compounds of methyl acetate, (E)-hex-2-enal, benzaldehyde, 5-ethyloxolan-2-one were considered as the common characteristics of SD and BJ, whereas [(Z)-hex-3-enyl] acetate was determined as the particular compound for BJ with the *p*(corr) close to 0 for SD.

The box plots of the five critical aroma compounds are demonstrated in Figure S3. The concentration of two compounds of methyl acetate, (E)-hex-2-enal and benzaldehyde were significantly higher in SD with the concentration of 602.58, 2688.33, and 4858 µg/kg (Figure S3a–c). As seen in Figure S3d, the concentration of [(Z)-hex-3-enyl] acetate was remarkably higher in BJ, being 1567.83 µg/kg compared with HB and SD. Moreover, 5-ethyloxolan-2-one was significantly higher in HB compared to BJ and SD with a concentration of 384.83 µg/kg (Figure S3e). In conclusion, as shown in the SUS plots and box-plots, the five compounds of methyl acetate, (E)-hex-2-enal, benzaldehyde, [(Z)-hex-3-enyl] acetate, and 5-ethyloxolan-2-one were identified as the critical markers for peach geographical origin traceability in BJ, HB, and SD.

### 3.4. Critical Aroma Compounds for Qualitative Discrimination Model and Potential Chemical Basis of Odors via Sensory Evaluation

#### 3.4.1. Critical Aroma Compound: Qualitative Discrimination Model Based on PLS-DA

The five critical aroma compounds of methyl acetate, (E)-hex-2-enal, benzaldehyde, [(Z)-hex-3-enyl] acetate, and 5-ethyloxolan-2-one were characterized as critical components by taking both the OAV and chemometrics methods into account. It is universally acknowledged that the PLS-DA approach is powerful in dealing with multi-classification problems; therefore, the qualitative analysis model for BJ, HB and SD was developed by the PLS-DA algorithm with the five critical aroma compounds to investigate whether the three cultivation regions could be distinguished using the determined primary compounds through the multi-classification model.

**Figure 4:**
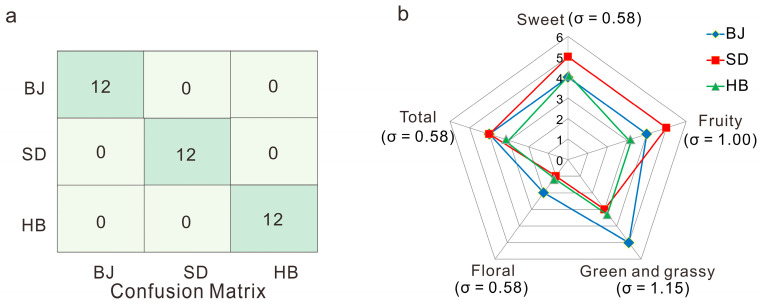
(**a**) The confusion matrix of PLS-DA model; (**b**) the sensory evaluation of the aroma profiles of peach from three cultivation regions of BJ, SD, and HB.

The first two latent variables (LVs) were selected to develop the multi-classification model according to the seven-fold cross-validation procedure with the total covariance of 0.972. As a result, the model developing ability of R^2^(Y) and predicted ability of Q^2^(Y) were 0.954 and 0.951, respectively. As obtained from the confusion matrix (Figure 4a), all the samples were located on the main diagonal, the classification accuracy for the three cultivation regions were all 100%. The PLS-DA model gained a favorable classification capacity according to accuracy, which was sufficient to suggest that the PLS model could feasibly distinguish the three cultivation regions using the five critical markers.

To sum up, the five critical markers acquired from the shared compounds from OAV and chemometrics methods were enough to develop the PLS-DA model for discriminating the three cultivation regions. Moreover, it can be concluded that the screened markers were overwhelmingly practicable to enable geographical origin traceability. It was also sufficient to indicate the marker explored methods of OAV, coupled with chemometrics approach, work effectively.

#### 3.4.2. Critical Aroma Compound: Potential Chemical Basis of Odors by Sensory Evaluation

The peach samples were used for a sensory evaluation in order to produce an overview of the flavors prevalent among the three cultivation regions. The standard deviation (σ) was applied to assess the stability of the peach flavor. In general, a larger σ indicates a greater difference in flavor. As demonstrated in Figure 4b, the ‘sweet’, ‘fruity’, ‘green and grassy’ odors were considered as the dominating aroma in peach. Among the four typical aroma attributes, ‘sweet’ and ‘floral’ exhibited the minimum variation (both with σ = 0.58), whereas, the σ for ‘green’ (σ = 1.15) and ‘grassy’ (σ = 1.00) were relatively larger. The intensity of the ‘sweet’ and ‘fruity’ odor in SD was stronger when compared with BJ and HB, which might have resulted from higher concentrations of methyl acetate, hexyl acetate, and benzaldehyde (Table 1). The ‘floral’ odor in BJ might be due to the high concentration of nonan-1-ol, whose concentration in BJ was three and four times higher than that in SD and HB. In addition, the peach in BJ exhibited the highest intensity of ‘green- and grassy-like’ odor, which might be due to the high concentration of [(Z)-hex-3-enyl] acetate (1567.83 µg/kg) compared with HB (524.58 µg/kg) and SD (482.25 µg/kg). Accordingly, BJ and SD had richer odor characteristics in contrast with HB.

As a consequence, it can be concluded that the sensory evaluation results might be closely related to the five screened potential critical aroma compounds with respect to the main flavors of ‘sweet’, ‘fruity’, ‘green and grassy’.

## 4. Conclusions

In this study, the volatile profile of peach was characterized using HS-SPME/GC-MS. Five aroma compounds of methyl acetate, (E)-hex-2-enal, benzaldehyde, [(Z)-hex-3-enyl] acetate, and 5-ethyloxolan-2-one were identified as differentially critical volatile components for the three cultivation regions of BJ, HB, and SD by taking into account both the primary aroma-active compounds with OAV > 1 and the critical compounds explored through chemometrics methods. The potentially significant compounds found using chemometrics methods were determined on the basis of significant difference (*p* < 0.05), FC, S-plot analysis, jack-knifing confidence interval, VIP, and the SUS plots. As visualized in the SUS-plots, the critical compound of [(Z)-hex-3-enyl] acetate was identified as the unique feature for BJ, whereas the compounds of methyl acetate, (E)-hex-2-enal, benzaldehyde, and 5-ethyloxolan-2-one were ascertained as the shared features. Moreover, the multi-classification model gave an outstanding performance, with an accuracy of 100%, and enabled the three cultivation regions to be distinguished using the five critical markers. The sensory evaluation results might be closely related to the five explored critical aroma compounds with respect to the main flavors of ‘sweet’, ‘fruity’, ‘green and grassy’. This research on potential aroma is extremely important for geographical origin traceability. Moreover, it also creates a guide to evaluate peach quality and provides a basis for understanding the formation of the characteristic aroma compounds in peaches

## Figures and Tables

**Table 1:** The volatile compounds of peach from three cultivation regions of BJ, SD, and HB. *nd* represents not detected.

No.	Volatile Compounds	RT (min)	Concentration (μg/kg)		
			BJ	SD	HB
1	Isocyanic acid	4.47	51.92 ± 6.52	87.67 ± 5.35	92.08 ± 7.68
2	Methyl acetate	4.93	123.67 ± 12.57	602.58 ± 86.40	97.50 ± 8.99
3	2-Methylpropane	5.97	4.83 ± 0.83	*nd*	*nd*
4	Pentan-3-one	6.45	13.17 ± 1.70	*nd*	*nd*
5	2-Butan-2-yloxybutane	8.39	129.67 ± 11.48	116.92 ± 23.45	188.00 ± 10.82
6	1-(Furan-2-yl)pentan-1-one	8.73	3.67 ± 0.98	14.00 ± 1.86	6.08 ± 1.62
7	(E)-Hex-2-enal	9.72	606.17 ± 52.16	2688.33 ± 331.83	65.75 ± 8.85
8	5-Methylhept-3-yne	9.77	*nd*	5.33 ± 1.15	*nd*
9	Ethylbenzene	9.85	166.08 ± 15.37	87.00 ± 7.83	88.83 ± 7.32
10	1,2-Xylene	10.18	264.67 ± 35.01	339.33 ± 46.64	425.92 ± 16.56
11	Methyl hexanoate	11.24	*nd*	*nd*	45.00 ± 7.77
12	Benzaldehyde	12.42	1546.42 ± 203.31	4858.00 ± 178.88	3628.92 ± 266.85
13	(2R)-2-Hydroxy-3-methylbutanenitrile	12.66	*nd*	*nd*	12.08 ± 1.62
14	[(Z)-Hex-3-enyl] acetate	13.25	1567.83 ± 248.83	482.25 ± 95.83	524.58 ± 98.45
15	Hexyl acetate	13.40	1915.08 ± 161.47	2290.92 ± 144.76	1782.25 ± 294.10
16	(Z)-Hex-2-enyl acetate	13.45	1821.17 ± 185.55	1826.42 ± 161.33	1592.25 ± 308.70
17	(E)-Hex-3-en-1-yne	13.75	7.67 ± 1.56	*nd*	*nd*
18	Phenylmethanol	14.27	*nd*	196.42 ± 12.44	*nd*
19	5-Ethyloxolan-2-one	14.49	135.50 ± 32.10	11.92 ± 3.29	384.83 ± 45.06
20	Octan-1-ol	14.85	35.67 ± 8.97	*nd*	*nd*
21	Nonan-1-ol	17.19	143.42 ± 26.49	44.58 ± 6.29	38.17 ± 10.31
22	[(E)-Hex-2-enyl] 3-Methylbutanoate	18.79	*nd*	28.00 ± 3.30	*nd*
23	2,3-Dimethylbut-3-en-2-ol	21.43	*nd*	15.42 ± 2.61	*nd*

**Table 2:** Odor description, odor threshold value of the identified volatiles in peach from three cultivation regions of BJ, SD, and HB in northern China.

No.	Volatile Compounds	RI ^a^	RI ^b^	Odor Description ^c^	Threshold (μg/L) ^d^	ID ^e^	Category
1	Isocyanic acid			/	*n.f*	MS, RI	acid
2	Methyl acetate			sweet, fruity	3	MS, RI, S	ester
3	2-Methylpropane	605	-	/	*n.f*	MS, RI, S	hydrocarbon
4	Pentan-3-one	703	700	ethereal acetone	40	MS, RI	ketone
5	Sec-Butyl ether	801	-	/	*n.f*	MS, RI, S	ethers
6	1-(2-Furyl) pentan-1-one	815	-	/	*n.f*	MS, RI	ketone
7	(E)-Hex-2-enal	858	854	green, leaf	30	MS, RI, S	aldehyde
8	5-Methylhept-3-yne	860	-	/	*n.f*	MS, RI	hydrocarbon
9	Ethylbenzene	863	864	/	1200	MS, RI	benzene
10	1,2-Xylene	877	-	geranium	450	MS, RI, S	benzene
11	Methyl hexanoate	922	924	fruity	70	MS, RI, S	ester
12	Benzaldehyde	970	970	sweet	750	MS, RI	aldehyde
13	(2R)-2-Hydroxy-3-methylbutanenitrile	980	-	/	*n.f*	MS, RI	alcohol
14	[(Z)-Hex-3-enyl] acetate	1004	1009	green, leaf	8	MS, RI	ester
15	Hexyl acetate	1011	1010	fruity	10	MS, RI, S	ester
16	(Z)-Hex-2-enyl acetate	1013	1005	/	*n.f*	MS, RI, S	ester
17	3-Hexen-1-yne	1025	-	/	*n.f*	MS, RI	hydrocarbon
18	Phenylmethanol	1047	1042	floral, rose	2500	MS, RI, S	alcohol
19	5-Ethyloxolan-2-one	1056	1056	sweet, coconut	50	MS, RI	lactone
20	Octan-1-ol	1071	1068	green	130	MS, RI	alcohol
21	Nonan-1-ol	1171	1172	floral rose	50	MS, RI, S	alcohol
22	[(E)-Hex-2-enyl] 3-methylbutanoate	1242	1244	/	*n.f*	MS, RI	ester
23	2,3-Dimethylbut-3-en-2-ol	1366	-	/	*n.f*	MS, RI	alcohol

^a^ RI, retention indexes was calculated refereed to the retention time of C_7_–C_40_ n-alkanes under the same condition. ^b^ RI, retention indexes was found in the NIST Chemistry WebBook, SRD 69, URL: https://webbook.nist.gov/chemistry/cas-ser/ accessed on 26 November 2022. ^c^ Odor description found in the literature with database (Flavornet; The LRI and Odor Database). ^d^ Odor threshold values from (Burdock, 2010). ^e^ Identification method. MS, identification based on the NIST 2017 mass spectral database; RI, retention index; S, the compounds were identified using authentic standard compounds. *n.f*, data were not found in the literature.

**Table 3:** The volatile compounds with OAVs > 1 in peach from three cultivation regions. OAV, odor activity value. BJ, SD, and HB represent Beijing, Shandong and Hebei provinces, respectively.

Volatile Compounds	OAV Values		
	BJ	SD	HB
Methyl acetate	41.22	200.90	32.50
(E)-Hex-2-enal	20.21	89.61	2.19
Benzaldehyde	2.06	6.48	4.84
[(Z)-Hex-3-enyl] acetate	195.98	60.28	65.60
Hexyl acetate	191.51	229.10	178.00
Nonan-1-ol	2.87	0.89	0.76
5-Ethyloxolan-2-one	2.71	0.24	7.70

**Table 4:** OPLS-DA model parameters for the three cultivation regions of BJ, SD, and HB. R^2^p(X) represents the variance related to class separation, R^2^(X) represents the total explained variance, R^2^(Y) represents the model developing ability, Q^2^(Y) represents the cross-validated predictive ability.

Modeling Parameters	R^2^p(X)	R^2^(X)	R^2^(Y)	Q^2^(Y)	Components	Intercept R^2^
BJ vs. SD	0.737	0.861	0.988	0.983	1 predictive + 1 orthogonal	0.078
BJ vs. HB	0.680	0.846	0.980	0.969	1 predictive + 1 orthogonal	0.058
SD vs. HB	0.641	0.729	0.994	0.984	1 predictive + 1 orthogonal	0.126

## Data Availability

All data presented within the article are available upon reasonable request from the corresponding authors.

## Supplementary Materials

The following supporting information can be downloaded at: https://www.mdpi.com/article/10.3390/foods12040837/s1. Figure S1: The score plot of PCA analysis of BJ, SD, and HB. Figure S2: Raw data of the uncertain compounds with larger confidence intervals. (a) 1,2-xylene in the model of BJ vs. HB; (b) benzaldehyde in the model of SD vs. HB; (c) phenylmethanol in the model of SD vs. HB. Figure S3: The box plots of the five critical aroma compounds. (a) methyl acetate; (b) (E)-hex-2-enal; (c) benzaldehyde; (d) [(Z)-hex-3-enyl] acetate; (e) 5-ethyloxolan-2-one. Table S1: The screened critical aroma compounds for pair-wise comparisons of BJ, SD, and HB.
