# Supplementary material for: Flavoromics Approach in Critical Aroma Compounds Exploration of Peach: Correlation to Origin Based on OAV Combined with Chemometrics
Source: Foods. 2023 Feb 16;12(4):837. doi: 10.3390/foods12040837 (PMC9957197; doi:10.3390/foods12040837)
Supplement: Supplementary file 1 [file foods-12-00837-s001.zip › foods-2091930-supplementary.pdf]

Supplementary Materials

Flavoromics Approach in Critical Aroma Compounds Exploration of Peach: Correlation to Origin Based on OAV Combined with Chemometrics

Qianqian Li [1], Bei Li [2], Rong Zhang [1], Shuyan Liu [1], Shupeng Yang [1], Yi Li [1] and Jianxun Li [1,*]

[1] Key Laboratory of Agro-products Quality and Safety Control in Storage and Transport Process, Ministry of Agriculture and Rural Affairs, Institute of Food Science and Technology, Chinese Academy of Agricultural Sciences, Beijing 100093, China
[2] Key Laboraory of Tropical Fruits and Vegetables Quality and Safety for State Market Regulation, Hainan Institute for Food Control, Haikou 570314, China
* Correspondence: lijianxun@caas.cn

Figure S1 The score plot of PCA analysis of BJ, SD, and HB.

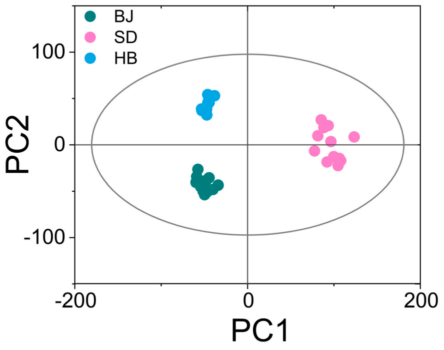

Figure S2 Raw data of the uncertain compounds with larger confidence intervals. (a) 1,2-xylene in the model of BJ vs HB; (b) benzaldehyde in the model of SD vs HB; (c) phenylmethanol in the model of SD vs HB.

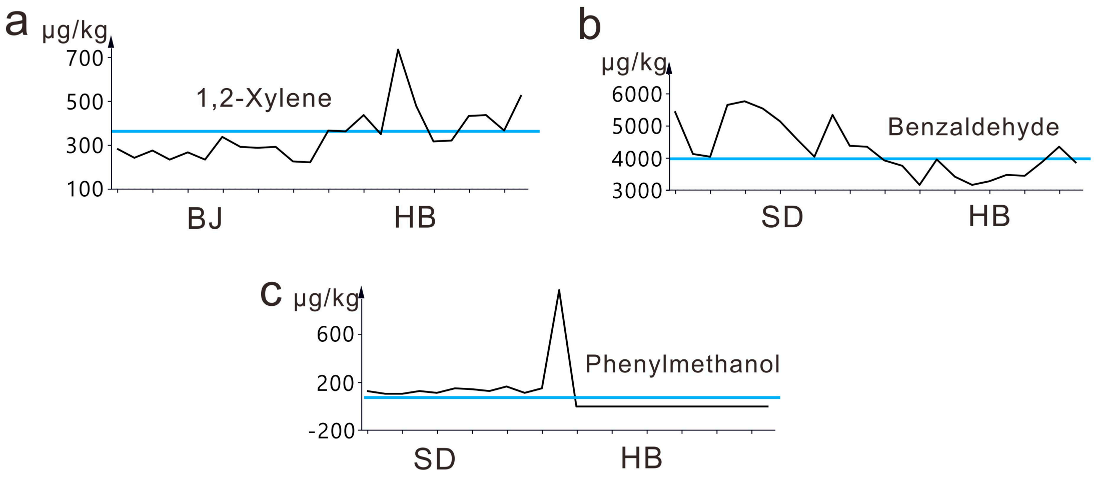

Figure S3 The box plots of the five critical aroma compounds. (a) methyl acetate; (b) (E)-hex-2-enal; (c) benzaldehyde; (d) [(Z)-hex-3-enyl] acetate; (e) 5-ethyloxolan-2-one.

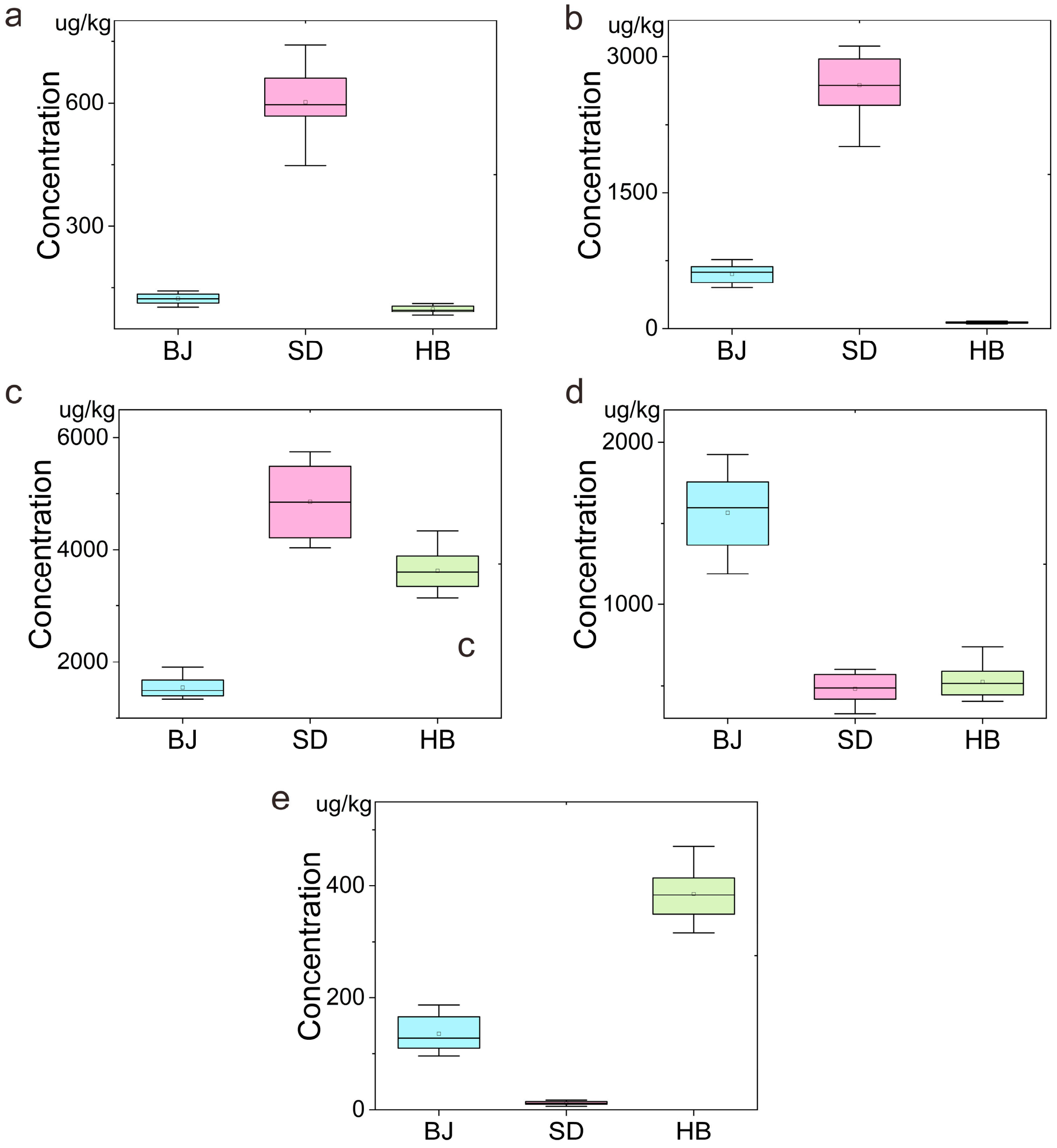
a
ug/kg
600
300
Concentration
BJ
SD
HB
b
ug/kg
3000
1500
0
Concentration
BJ
SD
HB
c
ug/kg
6000
4000
2000
Concentration
c
BJ
SD
HB
d
ug/kg
2000
1000
Concentration
BJ
SD
HB
e
ug/kg
400
200
0
Concentration
BJ
SD
HB

Table S1 The screened critical aroma compounds for pair-wise comparisons of BJ, SD, and HB.

Models	Compounds (VIP>1)	VIP value	FC	*p* value	Critical aroma compounds
BJ vs SD	Methyl acetate	1.16	4.87	5.04E-10	Methyl acetate, (E)-Hex-2-ene, Benzaldehyde, [(Z)-Hex-3-enyl] acetate
	(E)-Hex-2-ene	2.41	4.43	1.82E-11	
	Benzaldehyde	3.08	3.14	5.63E-10	
	[(Z)-Hex-3-enyl] acetate	1.72	0.31	9.60E-10	
BJ vs HB	(E)-Hex-2-ene	1.65	9.22	1.76E-9	(E)-Hex-2-ene, Benzaldehyde, [(Z)-Hex-3-enyl] acetate, 5-Ethyloxolan-2-one
	Benzaldehyde	3.31	0.43	2.91E-12	
	[(Z)-Hex-3-enyl] acetate	2.30	0.35	2.19E-13	
	5-Ethyloxolan-2-one	1.12	2.99	1.47E-9	
SD vs HB	Methyl acetate	1.44	6.18	3.58E-10	Methyl acetate, (E)-Hex-2-ene, 5-Ethyloxolan-2-one
	(E)-Hex-2-ene	3.30	40.89	1.76E-11	
	Benzaldehyde	2.02	1.34	3.80E-5	
	5-Ethyloxolan-2-one	1.26	0.03	9.20E-12	
